# Exploring the Users’ Perspective of the Nationwide Self-Exclusion Service for Gambling Disorder, “Spelpaus”: Qualitative Interview Study

**DOI:** 10.2196/66045

**Published:** 2025-01-31

**Authors:** Johanna Tjernberg, Sara Helgesson, Anders Håkansson, Helena Hansson

**Affiliations:** 1 Department of Clinical Sciences Lund Faculty of Medicine Lund University Lund Sweden

**Keywords:** gambling disorder, gambling addiction, behavioral addiction, harm reduction, self-exclusion, voluntary self-exclusion, Spelpaus, lived experience, human factors, usability, qualitative study

## Abstract

**Background:**

Problem gambling and gambling disorder cause severe social, psychiatric, and financial consequences, and voluntary self-exclusion is a common harm reduction tool used by individuals with gambling problems.

**Objective:**

The aim of this study was to explore users’ experience of a novel nationwide, multioperator gambling self-exclusion service, “Spelpaus,” in Sweden and to inform stakeholders and policy makers in order to improve harm reduction tools against gambling problems.

**Methods:**

Semistructured interviews were conducted with 15 individuals who reported self-perceived gambling problems and who had experience of having used the self-exclusion service Spelpaus in Sweden. Interviews were transcribed and analyzed through qualitative content analysis.

**Results:**

We identified 3 categories and 8 subcategories. The categories were (1) reasons for the decision to self-exclude, (2) positive experiences, and (3) suggestions for improvement. The subcategories identified a number of reasons for self-exclusion, such as financial reasons and family reasons, and positive experiences described as a relief from gambling; in addition, important suggestions for improvement were cited, such as a more gradual return to gambling post–self-exclusion, better ways to address loopholes in the system, and transfer from self-exclusion to treatment.

**Conclusions:**

Voluntary self-exclusion from gambling, using a nationwide multioperator service, remains an appreciated harm-reducing tool. However, transfer from self-exclusion to treatment should be facilitated by policy making, and loopholes allowing for breaching of the self-exclusion need to be counteracted.

## Introduction

### Background

Gambling disorder is a behavioral addictive disorder known to cause severe financial, social, and mental health consequences to affected individuals [1-3]. Among the harm-reducing or harm-preventive strategies used against gambling-related harm, voluntary self-exclusion is a well-established responsible gambling measure aiming to prevent and reduce the damage that can be caused by gambling [4].

Through self-exclusion, gamblers can voluntarily suspend themselves from gambling. However, 1 well-known limitation of self-exclusion services may be the possibility to gamble with another operator than the one the gambler has been self-excluded from [5]. Gamblers are therefore usually required to self-exclude from multiple different operators. Consequently, in recent years, to further prevent gamblers from breaching their self-exclusion, it has become more common to offer gamblers the possibility to self-exclude from multiple operators at the same time.

In January 2019, the legislation for the Swedish gambling market changed, and monopoly was replaced by a license-regulated market [6]. This regulation meant that gambling operators, including overseas operators, needed to apply for a Swedish gambling license to operate on the Swedish market. In connection to the new regulations, the nationwide self-exclusion service “Spelpaus” was launched. Gambling operators with a Swedish license must adhere to this self-exclusion service, along with a series of other measures for responsible gambling. Spelpaus offers the possibility to self-exclude from all gambling operators with a Swedish license for 1, 3, 6, or 12 months. Self-exclusion cannot be canceled prematurely, and if the 12-month option is chosen, the self-exclusion continues beyond the 12 months, unless the individual actively chooses to cancel it after that period. Self-exclusion via Spelpaus also prohibits gambling operators from sending direct advertisements to self-excluded customers.

Spelpaus is in many ways a unique harm-reducing tool since it is nationwide, covers all licensed gambling operators (both online and land based), and can be accessed separately from the gambling operators. Thus, it theoretically represents a novel harm reduction strategy over and above other responsible gambling measures. Indeed, theoretically, this system may have a larger coverage and fewer loopholes (ie, fewer practical possibilities to turn into other gambling modalities and gamble despite self-exclusion) compared to a self-exclusion system involving 1 or few gambling operators. Around 100 gambling companies have a Swedish gambling license, with most of them operating exclusively online.

The prevalence of gambling problems in the Swedish population is estimated to be approximately 0.5% on a diagnostic level and a total of 1%-2% with at least moderate-risk gambling according to the Problem Gambling Severity Index [7,8]. Formal treatment seeking, or treatment provision, is low; less than 1000 people annually receive a gambling disorder diagnosis in the whole of Sweden, and even when taking other care providers into considerations, it can be assumed that only a small minority of people with a gambling problem on a diagnostic level receive formal treatment [9]. In contrast, low formal treatment seeking is often described in gambling disorder; however, a more informal but still active help-seeking behavior appears to be common, and voluntary self-exclusion from gambling can be part of this [10].

In Sweden, around 110,000 inhabitants are enrolled in Spelpaus [11], a number that has steadily increased since the launch in 2019 [12]. Thus, we believe that an increasing number of people with problematic gambling are attempting to intervene against their problem through self-exclusion, although we must consider that people without gambling problems also choose to self-exclude.

The steady increase in registrations in Spelpaus indicates that it is perceived as a helpful tool for many people. However, there are also limitations in the self-exclusion service, making it possible to breach the self-exclusion. Such a limitation is that Spelpaus only covers operators with a Swedish license, therefore making it possible for gamblers to gamble with overseas operators outside the Swedish license gambling market. In a 2020 web survey among gamblers, 38% of gamblers stated that they had gambled with unlicensed operators, while being self-excluded through Spelpaus [13], and in a later survey in 2022, the corresponding figure was 49% [14]. In another survey, conducted by the Swedish Gambling Authority, 25% gamblers stated that they had gambled with unlicensed operators during their self-exclusion [15]. In addition, continued gambling despite self-exclusion remains part of the clinical picture in clinical gambling disorder treatment in this setting [16].

Previous international experience of responsible gambling tools has demonstrated not only the feasibility of self-exclusion services and their use by individuals with gambling problems [4,17,18] but also that users experience limitations of these services [10,18]. Altogether, previous research calls for more in-depth examinations of user experience with self-exclusion services and suggestions for improvement of their effectiveness. In particular, this is relevant for the present type of self-exclusion service, which theoretically has been designed to overcome well-known limitations of older self-excluson systems; this novel Swedish service (1) is government based and independent of gambling operators, such that an individual does not need to enter a gambling site in order to self-exclude; (2) covers all licensed operators in the country and therefore prevents the possibility to change between licensed operators during an ongoing self-exclusion period; and (3) involves both land-based and online-based operators.

Thus, altogether, the relatively recent introduction of a unique, large-scale, multioperator self-exclusion service in Sweden has attracted a large number of users, but it is hindered by the risk of breaching one’s self-exclusion to an extent that is clearly of relevance in the treatment setting. Therefore, it is of great value to study which effects of the Spelpaus service are perceived as favorable and challenging by its users and to also study this in more detail than in previous quantitative survey studies.

### Aim

Based on the aforementioned research gap, the aim of this study was to enhance comprehension of the user experience of the Spelpaus service within the gambling community and to acquire a more profound insight into the reasons for use of this harm reduction tool and its advantages and challenges. Thereby, the overarching goal of the study was to inform policy makers about potential drawbacks and the potential for improvement of the harm reduction tool.

## Methods

### Study Design

This research was exploratory in nature. A semistructured interview guide was developed to ensure the exploration of diverse perspectives and experiences related to Spelpaus. Notably, this study represents the first attempt to comprehensively examine user experiences of Spelpaus, thus justifying the use of qualitative content analysis for its methodological flexibility. The protocol of this study has been published previously [19].

An initial interview guide was developed based on the study’s aim and research questions. A test interview with a peer support worker who had experience with self-exclusion services was conducted, leading to refinements of some questions to be more open ended. Further details of the interview questions are provided in the study protocol [19].

### Context

The study was conducted in Sweden, a country with approximately 10.4 million inhabitants, where gambling is prevalent, with around 56% of individuals aged from 16 to 84 years having engaged in gambling activities in the past year. Moreover, 18% gamble at least once per month. The prevalence of gambling is higher among men aged 45-84 years, and online gambling has increased markedly in popularity in Sweden over the past decade [20]. Problem gambling in the present setting is predominated by online casino gambling and sports betting, with online casino gambling representing a large majority of patients seeking treatment at a gambling disorder facility in Sweden [16].

Sweden has a license gambling market since 2019, where online and land-based gambling operators are allowed to operate, provided they adhere to a number of responsible gambling regulations, one of which is adherence to the nationwide Spelpaus service. The legal gambling age in Sweden is 18 years [6]. After being a more traditional, land-based gambling market, but with a large predominance of a government-owned gambling monopoly for many years [21], the Swedish gambling market was gradually increasingly affected by overseas online operators, which represented a large proportion of visible gambling advertising despite their unregulated status [22]. This led to the decision to liberalize the gambling market into a licensed, but controlled, market from 2019. Given the new license-based gambling market since 2019, and thereby the relative recency of a unique self-exclusion service, the present setting is of interest to assess the qualities and challenges of such a new harm reduction instrument.

Potential stakeholders and users of the study’s findings may include policy makers, public health officials, mental health professionals, gambling addiction counselors, researchers in the field of addiction studies, advocacy groups, and individuals and families affected by problem gambling. The insights garnered from this research could inform the development of targeted interventions, public health campaigns, regulatory measures, and support services aimed at addressing and mitigating the adverse effects of gambling behavior within the Swedish population.

### Recruitment Process

Criterion sampling was used in the recruitment process. Participants were selected based on 2 critera: (1) being 18 years old or older during the time of the interview and (2) having previous or ongoing experience of using Spelpaus (any experience was enough, and it was not a requirement that the self-exclusion be in close temporal association with the interview). Recruitment was conducted online through social media advertisements managed by Trialy, a company specializing in research recruitment. Potential participants received information about the study and registered their interest via the Trialy website, completing a short online survey regarding their age and experience with Spelpaus. Eligible individuals were contacted by the first author (JT) and provided with additional study information. They were then sent written information about the study and about participants’ rights and asked to sign and return (by mail) an informed consent form. When written consent was obtained, an interview was scheduled. Individuals were recruited during spring-summer 2023.

### Sample

In total, 13 men and 2 women, aged between 31 and 62 years, participated in the study, representing various regions of Sweden. All participants reported either a gambling problem or identified themselves as being at risk, with prior or current experience using the Spelpaus self-exclusion service. This gender distribution aligned with existing research findings on gambling demographics in Sweden, which indicate a higher prevalence of gambling-related issues among men [16]. The gambling types involved in each person’s gambling pattern were reported. Although the study did not systematically record whether gambling occurred (and had occurred) in online-based or land-based settings, the distribution of gambling types reported by the participants was comparable with the gambling types typically seen in Swedish problem gambling treatment settings, where online casino and sports betting is predominant [16].

### Interviews

All interviews were digital (both video and audio) and were carried out by the first author (JT) of the paper, who has experience in qualitative interviewing and psychiatric research, especially in psychiatric research, ensuring a sensitive and nuanced data collection approach. The interviews were audiorecorded but not videorecorded and lasted between 23 and 67 minutes. During the interview process, the authors continuously reviewed and assessed the interviews to discern recurring patterns. Following the completion of 12 interviews, the emergence of new categories began to decline. In the final 2 interviews, only categories closely aligned with those identified previously surfaced. Consequently, the authors made the decision to conclude recruitment for the study. All interviews were then transcribed verbatim by the first and second authors (JT and SH).

As compensation for their time and efforts in the study, all participants received 2 cinema tickets.

### Researcher Characteristics and Reflexivity

Our research team included professionals in psychiatry and social work, with substantial experience in qualitative research and interviewing. Throughout the study, we reflected on our backgrounds and preconceptions, particularly regarding gambling disorders and harm reduction strategies, and their potential influence on data interpretation. This reflexivity was integrated into our coding and analysis discussions to include multiple perspectives and thereby to expand our own perspectives.

To ensure trustworthiness, triangulation was used throughout the analysis process. Multiple researchers independently coded the data, followed by discussions to reach consensus, minimizing the influence of individual biases and ensuring comprehensive data representation.

### Data Analysis

This study used qualitative content analysis as the primary method for data analysis, chosen for its flexibility in systematically describing textual data, aligning well with the exploratory nature of this research. The analysis focused on manifest content, which involves identifying clear, descriptive categories that summarize data without deeper interpretive meanings. As noted by Graneheim and Lundman [23], categories represent the descriptive level of content and express the manifest content of the text, consistent with this study’s goals. We deliberately chose not to identify themes, aiming instead to capture the descriptive aspects of participants’ experiences, a recommended approach when focusing on manifest content rather than latent content [24,25]. Although themes can provide interpretative layers, we chose not to use them in this analysis, maintaining a descriptive focus to capture clear, observable patterns in participants’ experiences with Spelpaus. This approach reflects the strength of qualitative content analysis in emphasizing the descriptive aspect without requiring thematic depth when the aim is to focus on manifest content [23].

The qualitative content analysis followed Graneheim and Lundman’s [23] approach, focusing on identifying categories that represented the manifest content of participants’ responses. Key analysis steps included repeated readings of the transcripts by 3 authors (JT, SH, and HH), identifying meaning units, condensing those units, and labeling them with codes, which were then grouped into categories based on similarities. The transcribed interviews were read through repeatedly and independently, and then the material was discussed by all authors. The content of the interviews related to the aim of the study was divided into meaning units, which were then condensed and labeled with codes. Similarities and differences in the codes were identified before they were divided into different categories and subcategories (Table 1). All steps of the analysis process were carried out separately and then discussed within the group to reach consensus. Various meetings within the group were held during the analysis process to discuss the different findings before a consensus about the results was reached.

**Table 1:** Examples of the data analysis process.

Meaning unit	Condensed meaning units	Code	Category	Subcategory
“If you should have a gambling stop like this, it should really apply to everything, because the brain will always find a way otherwise. This is the good thing—that it is a stop. You can’t make a phone call, you can’t just change your mind, because now it is a stop. I think that is very important.” [Male participant, 31 years old]	The gambling stop should really apply to everything. Because the brain will always find a way otherwise. The good thing is that it is a stop, you cannot just change your mind. I think that is important.	Satisfaction with Spelpaus	Positive experiences	Prevention of future gambling problems
“Well, the downside is that it gives a little bit of a false sense of security. At least when it comes to certain people...as if you gamble on other gambling sites...it doesn’t help anything.” [Female participant, 35 years old]	The downside is that it gives a little bit of a false sense of security. When it comes to certain people who gamble on other sites, it does not help anything.	Suggestion for improvement: to also stop overseas gambling sites	Suggestions for improvement	Addressing loopholes

### Ethical Considerations

The study was conducted according to the principles of the Declaration of Helsinki and was approved by the Swedish Ethical Review Authority (approval number 2022/06933-01, amendment 2023/01684-02). All participants received oral and written information about the study and about participants’ rights, prior to signing a consent form. Written informed consent was obtained from all participants.

## Results

### Participant Details

A detailed description of the 15 participants is presented in Table 2.

**Table 2:** Background information about the participants (N=15).

Characteristics		Participants, n (%)
**Social relationships**		
	In a relationship	8 (53)
	Have children	9 (60)
**Gambling form**		
	Casino	3 (20)
	Sports betting	1 (7)
	Horse race betting	0
	Lottery	1 (7)
	Casino + sports betting	2 (13)
	Casino + poker	3 (20)
	Casino + horse race betting	1 (7)
	>2 different gambling forms	4 (27)
**Enrolled in Spelpaus at the time of the interview**		
	Yes	10 (67)
	No	3 (20)
	N/A^a^	2 (13)
**In active gambling at the time of the interview**		
	Yes	3 (20)
	No	11 (73)
	N/A	1 (7)
**Number of times using Spelpaus**		
	1	4 (27)
	≥2	11 (73)
**Chosen time interval of self-exclusion**		
	12 months	7 (47)
	1, 3, or 6 months	3 (20)
	Both of the above	5 (33)
**Overseas gambling during Spelpaus**		
	Yes	6 (40)
	No	9 (60)

^a^Not applicable.

### Categories and Subcategories

As shown in Table 3, we identified 3 categories: motivations and reasons for self-exclusion (including triggers, such as financial strain and concern for close relationships), perceived positive experiences (eg, reduced gambling urges and improved quality of life), and challenges and suggestions for improvement (notably, breaches through unlicensed gambling sites and lack of integrated support).

**Table 3:** Categories and subcategories.

Category	Subcategories
Reasons for the decision to self-exclude	Precipitating factors Self-awareness of gambling habits Concern for close ones
Positive experiences	Prevention of future gambling problems Improved quality of life
Suggestions for improvement	Integrated support systems Addressing loopholes Practical enhancements

#### Reasons for the Decision to Self-Exclude

The motivations underlying the choice to use the self-exclusion service were multifaceted, often encompassing a combination of factors.

#### Precipitating Factors

Numerous participants reported specific events that precipitated heightened gambling activity, ultimately leading to the decision to self-exclude. Common triggers included winning a large amount of money and quickly gambling it away, traumatic family incidents, interpersonal conflicts, financial struggles, and the onset of the COVID-19 pandemic. For instance, 1 (7%) participant articulated:

I was facing unemployment without insurance, which exacerbated my gambling tendencies. Although I secured employment thereafter, the meager income did not deter my gambling. The resurgence of COVID-19 in September restarted my gambling habits, leading to a downward spiral.

Male participant, 44 years old

Additionally, some participants opted for self-exclusion to prevent the risk of others accessing their gambling accounts as part of gambling-related criminal behavior. An example of this was the following interviewee who had a gambling problem but who also had a family member with a severe gambling problem:

...At the same time, this has helped us a lot every time she has wished to gamble on our accounts.

Female participant, 35 years old

Another reason to self-exclude was because the individual wished to facilitate debt settlement, as self-exclusion was a prerequisite for debt relief applications.

I applied for a debt settlement, but since I did not take it [self-exclusion] for a year, I did not get it…I would like to make another attempt for a debt settlement application, but then I probably have to take a 12-month period [of self-exclusion] as well.

#### Self-Awareness of Gambling Habits

A prevalent reason for self-exclusion was an enhanced awareness of one’s gambling behavior. Participants acknowledged excessive gambling tendencies, albeit with varying perspectives on whether they had developed a full-fledged gambling problem or were teetering on the brink of one. Fueled by these insights, individuals opted for Spelpaus to forestall further progression into problem gambling or to address existing issues. As 1 (7%) participant reflected:

I have had a realization, particularly in recent months...I recognize that I need to address this behavior before it escalates.

Female participant, 56 years old

This introspective process often unfolded gradually, sometimes facilitated by professional guidance or peer support.

#### Concern for Close Ones

Many participants described the adverse impact of their gambling habits on loved ones, with 10 (67%) individuals mentioning that their loved ones had been negatively impacted to a great extent. Financial strain, deceit, and compromised relationships were common themes. Some participants lamented fractured relationships resulting from their gambling, while others acknowledged the toll gambling took on family bonds and quality time spent together. One participant expressed:

It is not just about the money lost—it is the betrayal, the lies...and the time squandered gambling instead of being with my family that truly stings.

Male participant, 39 years old

Although self-exclusion was often an independent decision, familial influence or shared deliberation with loved ones also played a role. The following 2 (13%) citations are examples of this:

The relationship with my parents is starting to get a little bit better; for many years, it was pretty bad. They have helped me out with money a lot of times. Every time I call them, they think I am going to ask them for money again, so it [gambling] has hurt our relationship a lot.

Male participant, 44 years old

If you fail in a game or something, you get kind of aggressive, depressed, and angry. You also get very affected emotionally. If you bet on 7 games and 1 of them fails, you get upset, angry, or sad. It has affected my close ones the most.

Male participant, 40 years old

#### Positive Experiences

Participants identified several benefits associated with using the Spelpaus self-exclusion service.

#### Prevention of Future Gambling Problems

Self-exclusion was perceived as a preemptive measure to safeguard against relapse into gambling behaviors. Even individuals who had not experienced gambling urges for an extended period recognized the potential for future vulnerability. By enacting self-exclusion, participants closed the door to impulsive gambling, thus mitigating the risk of relapse. Several participants credited the Spelpaus service for preventing immediate urges to gamble. A couple of the participants who began gambling again described how they after the end of the self-exclusion period managed to gamble in a more controlled way than before.

After the self-exclusion, it was much more under control. I gambled a lot less, bet smaller amounts…if I felt I need to limit it now or I have to stop now. When you feel it is starting to be too much or take too much time, you feel a little self-control. Then I take a free weekend or several days free from betting, and after that, I bet a little, and then it feels like everything is under control.

Male participant, 40 years old

Another participant described the partial effect on gambling, primarily on impulse-driven uncontrolled gambling:

A part of me does not really want to say goodbye and never gamble again, but I want it to be at a reasonable level. It is at ATG [Swedish horse-betting company]. I am staying there. I think I gamble for 96 (Swedish) kronor a week now, but it was still that break (self-exclusion) that forced me to deal with it somehow.

Male participant, 55 years old

#### Improved Quality of Life

Many participants reported enhanced well-being during the self-exclusion period. Freed from the grip of gambling, individuals experienced a newfound sense of calm, happiness, and control over their lives. Moreover, self-exclusion provided an opportunity for personal growth and self-improvement, allowing participants to redirect their focus toward constructive pursuits, such as physical exercise and social interactions. One individual reflected:

You can relax because you have given away the control to something else…once I have chosen to self-exclude, I cannot do anything about it…and I get so mentally relaxed, I do not have to think about it at all anymore.

Male participant, 32 years old

Financial stability and restored trust within familial relationships were also cited as positive outcomes, as 1 (7%) respondent expressed:

My family can tell that I feel better when I do not gamble. I am happier, more positive, and I do not call them and ask for help. Of course, they notice a big difference when I do not gamble.

Male participant, 31 years old

In addition, the self-exclusion period provided an opportunity for reflection within the motivational process:

When I had my Spelpaus, I became quite aware of the problem, so I had a lot of thoughts about the need to do something about this problem.

Male participant, 40 years old

#### Suggestions for Improvement

Participants identified several areas for enhancement within the Spelpaus self-exclusion service.

#### Integrated Support Systems

Acknowledging self-exclusion as 1 component of the multifaceted approach to gambling cessation, participants emphasized the need for additional support mechanisms. One participant expressed:

My fear is that when the self-exclusion disappears, I have not come so far that I will not get stuck in gambling again…I think it is important that you try to solve your problems when you are self-excluded.

Male participant, 43 years old

Peer support groups, counseling services, and psychotherapy were deemed vital complements to self-exclusion. However, participants expressed reluctance to seek out support independently, underscoring the importance of proactive outreach and integrated support systems from within Spelpaus. Some of the participants who considered themselves to be more at risk of developing a gambling problem expressed that Spelpaus alone was enough for them.

If you take a year, and you have the opportunity to end it, then there needs to be some kind of dialogue before, and some kind of amount limit.

Male participant, 55 years old

I think it should be combined with a guaranteed CBT [cognitive behavioural therapy] or diary where you write what you feel, what you can do and what you think you should be able to do. Then of course, in the best of worlds, group therapy is perhaps even better than being digital.

Male participant, 55, years old

#### Addressing Loopholes

Participants raised concerns regarding the efficacy of Spelpaus in preventing access to overseas gambling sites. The absence of coverage for nondomestic operators posed a significant loophole, enabling some individuals to circumvent self-exclusion measures. Suggestions for improvement included enhanced international cooperation and stricter regulations on gambling advertisements.

#### Practical Enhancements

Participants proposed various technical and logistical improvements to Spelpaus, including more flexible and expanded self-exclusion options, longer time intervals, and a more deliberative process for ending self-exclusion periods. Mainly, participants asked for time intervals longer than 12 months. Several participants saw it as problematic to be able to gamble immediately after the end of a self-exclusion period. Additionally, participants advocated for heightened demands on gambling operators to deter relapse and streamline the self-exclusion process.

The self-exclusion service should be able to be developed so you cannot access overseas sites…when you select it, it should be covering all sites within the EU [European Union] or something like that…that you have a self-exclusion service for all of Europe when you choose it.

Male participant, 62 years old

The best thing would have been if there was a self-exclusion site where you are self-excluded from all gambling…that you have some kind of cooperation with other countries and everything and that it will be a stop everywhere.

Female participant, 35 years old

Several respondents stated that the possibility of returning to gambling after the self-exclusion period should still be hindered or slowed down in order to prevent impulse-related relapses even after the predetermined self-exclusion period is over. Some examples of this was an “embargo” period during which gambling still could not be reopened. Some participants found themselves waiting to gamble immediately after the self-exclusion period ended, so they suggested there be some kind of deposit limit for all clients coming back from self-exclusion, a mandatory telephone call or other message to individuals for whom self-exckusion is ending, prohibition of direct advertising to individuals immediately after a self-exclusion period, or mechanisms making the risk of gambling during alcohol influence smaller (eg, through prohibition of gambling during the night).

One respondent described the need for an embargo period:

After 12 months, you should not be able to cancel it on the same day; it should be 1, 2, 3 months notice…otherwise, when 12 months have passed, you can just gamble without any restrictions.

Male participant, 32 years old

Other respondents described the need for other possible measures to apply in the policy of self-exclusion:

After the self-exclusion ended, the advertising started coming again. It becomes like a reminder. So one could simply get going and gamble again!

Male participant, 31 years old

There should be some time to think it over when a client has asked to come back to gambling again. Some time should elapse, and then the person should be asked again. After some time, you should be asked about whether you really want to gamble again. There are people who get a kick, but after some time, that feeling migh pass again.

Male participant, 44 years old

## Discussion

### Principal Findings

This study aimed to deepen the understanding of the user experience of a nationwide, multioperator self-exclusion service, beyond what is known from recent quantitative research studies with web panel members and clinical data from patients in treatment for a gambling disorder. The potential challenges of a multioperator self-exclusion service have been demonstrated in surveys, showing that primarily among individuals with highly intense gambling practices, self-exclusion is popular but often breached by its users [13,14]. However, advantages of being self-excluded, and a deeper understanding of how this is related to the challenges of the method, largely remain to be understood.

Here, this qualitative study resulted in the description of 3 overarching categories that provide a deeper understanding of the problem: the main reasons for the decision to self-exclude, the advantages perceived by the users of this method, and the users’ suggestions for improvements.

The decision to self-exclude from gambling platforms is often prompted by a sudden realization of escalating gambling behavior and a subsequent desire to regain control. Although the development of a disordered gambling pattern or financial difficulties were reported to have contributed to the decision to self-exclude, it was also reported, less expectedly, that self-exclusion may also be triggered by a wish to prevent another person from gambling on one’s account. This may be a less expected reason for self-exclusion and will merit further investigation, especially in relation to the role of gambling in criminal behavior. Gambling or conducting financial transactions using somebody else’s identity may represent one of the features of a severe gambling problem and also constitutes a criminal act [26].

Importantly, the role of the gambler’s concerned significant others emerged as one of the reasons for self-exclusion. This highlights the importance of further addressing the interplay between individuals with gambling problems and their close ones and may also facilitate treatment processes if families of the affected individual are actively involved. This finding is consistent with previous research indicating that concerned significant others can favor treatment seeking and a favorable treatment outcome in their near ones with gambling problems [27,28]. In addition, the active participation of partners in the treatment of patients with a gambling disorder can be beneficial, both to the outcome of the affected patients and to the well-being of patients and their partners [29]. Thus, altogether, concerned significant others appear to play an important role through different phases of help seeking in individuals with problem gambling, even in the earlier harm-reducing interventions that self-exclusion is meant to represent.

Furthermore, the data showed that one reason for self-exclusion may be the intention to demonstrate one’s willingness to quit gambling when formally applying for a public service where this is either required or believed to have an effect favoring the application process. This type of instrumental decision to self-exclude in order to obtain specific services or to fulfill expectations of external decision makers rarely has been reported in this context before. Whether this happens as part of an individual motivational process or entirely based on the requirements from others remains to be studied.

When external factors are cited as a reason to self-exclude, whether it be the concern of family members or the requirements from a treatment setting or authority, it can be argued that the choice to self-exclude does not represent the same decisive step in a motivational process as it does when an individual self-excludes based on their own decision to stop gambling in order to prevent or alleviate symptoms of addiction. Thus, the possibly different levels of personal commitment associated with self-excluding will be relevant to include in the theoretical framework of self-exclusion in the future.

Overall, many favorable effects from self-exclusion were cited, despite previous experience where research has highlighted some major limitations of this service. Previous studies have been conducted using quite different methodologies, relying on larger quantitative data sets from online surveys [13,14] and clinical documentation [16], and where the findings were primarily the high rates of breaching one’s self-exclusion within this service. This paper, importantly, adds to the literature regarding the present type of multioperator, nationwide self-exclusion service; in previous publications from this setting, it has been highlighted that the rate of breaching of one’s self-exclusion is high in people with a gambling disorder [16] or with intensive online gambling patterns [14]. Here, several respondents described a large favorable impact on life after having self-excluded. Therefore, the description of more favorable experiences from this study provides a valuable experience, which contributes to the overall picture of this model, and strongly deepens the understanding of how this service fairs in affected individuals, over and above previous analyses conducted with quantitative methodology.

Although self-exclusion offered a respite from gambling-related stressors, several suggestions for improvements of the system emerged from the interviews. Participants emphasized the importance of holistic support systems and regulatory measures to augment its efficacy as a harm reduction tool. Suggestions for improvement partly followed what could be expected from previous web survey studies on gamblers; as many individuals who self-exclude also struggle with the risk of relapsing on overseas and nonlicensed gambling sites, users desired further legislative efforts making it possible to also self-exclude from other services, including those that are registered outside the present jurisdiction. Gambling in unlicensed, overseas online casinos has been reported to be the most common means of breaching one’s self-exclusion period, as reported in the online surveys previously conducted here in this setting [13,14]. Thus, one major implication of this is the political process of how to exclude unlicensed gambling operators from the gambling market or to prevent financial transactions to gambling companies operating abroad. The legal framework and technical boundaries of this go beyond the scope of this study, but these findings highlight the need for gambling regulations to stretch beyond the controlling of the land-based gambling opportunity within each geographical jurisdiction.

One important and relatively novel suggestion was the linking between a mere harm-reducing self-exclusion service and actual therapeutic efforts against the addictive disorder. Importantly, this would move the purpose of the self-exclusion service from being an anonymous electronic support tool to actually providing a conduit to treatment. Treatment seeking in gambling disorder is known to be low, with several barriers reported [18]. Importantly, it has been reported that despite perceived barriers against treatment seeking, individuals with gambling problems may use more informal ways to seek help and not necessarily formal enrolment into psychotherapeutic treatment. For example, this may involve access to online advice or other online tools to facilitate a behavioral change [10]. In that aspect, any degree of referral from self-exclusion to different degrees of online support, advice, or treatment may be a way forward to improve the outcome of a person with gambling problems, over and above the effect of self-exclusion per se.

Such a system of transferring self-excluding clients to addiction treatment has not, to the best of our knowledge, been described in previous literature, and this may suggest a new area of research. For example, in such a system, it would have to be considered whether there is a potential barrier against enrolling in a self-exclusion service if it may, under some conditions, trigger a personal contact from a gambling operator, from a public authority, or from a treatment provider. However, it has been demonstrated that unsolicited motivational telephone calls to people displaying hazardous gambling habits may be a way to lower wagering and increase harm-reducing measures [30,31] and that acceptability of such motivational efforts appears to be high [32]. Thus, if self-exclusion from gambling would trigger an outreach effort aiming to improve the individual’s gambling, it could be argued that this would not require a formal therapist-guided treatment but possibly may involve a brief, personalized intervention but with the capacity to refer individuals to further treatment when needed. Such individualized, personalized normative feedback has been suggested to be effective in other treatment or harm reduction interventions in problem gambling [33,34] and can initiate or enhance an internal motivational process in affected individuals, over and above the effects from the sole self-exclusion.

Likewise, another expansion of the self-exclusion tool, as suggested by this study, would be to slow down the procedure of opening up gambling opportunities after the self-exclusion period is terminated. This would also be a novel strategy that would require further study, but potentially, given the loss of the control component in gambling disorder, a more gradual onset of gambling after the self-exclusion period may provide further possibility to consider one’s choice to gamble and may reduce the harm potentially associated with a sudden onset of high-risk gambling. Likely, any tool that slows down access to online gambling may potentially have a harm-reducing and relapse-preventing effect [35].

Overall, including the considerations made by interviewees in this study and their suggestions for improvement, a self-exclusion service generally appears to be promising and possible to increase even further. However, the major obstacle to this method, the risk of breaching one’s self-exclusion through the use of gambling sites outside of the system, may remain. Here, one also has to consider the fact that the choice to self-exclude may cause the gambling patterns upon a potential relapse to happen on an unlicensed market and thereby with a lower degree of control than inside a licensed market. Previous experience, however, has pointed out that in individuals with extremely intense gambling patterns, such gambling patterns may happen on many operators, and a limit set upon one’s gambling in one setting may be followed by gambling on another site. Still, even when this happens, such as during a deposit limit imposed during the COVID-19 pandemic in Sweden, still many clients report such a limit to be favorable and to have decreased their gambling pattern, rather than the opposite [36]. Thus, although a method such as self-exclusion cannot hinder all types of gambling, it may still have motivational effects that limit the money lost and limits some of the consequences even in a high-risk gambling behaviour.

### Strengths and Limitations

This study has a number of potential limitations, but given the novelty of the research topic and the merits of an in-depth qualitative study design, it also has considerable strengths. Recruitment via social media advertisements and digital interviews can be considered as both a strength and a limitation. The recruitment method enabled recruitment without any geographical limitations, therefore enabling a wide sample of individuals with different backgrounds, gambling habits, and experiences. However, it is possible that advertisements in social media attract interviewees with slightly different views on and experiences of self-exclusion from gambling than help-seeking individuals or individuals currently attending some type of land-based or online-based gambling venue. In contrast, the capacity of a qualitative design to identify a range of different aspects of a behaviour may benefit from this type of broad recruitment.

Digital interviews may represent another limitation, as these cannot readily be compared with physical interviews. Small changes in tone and body language are easily overlooked when the interview is held online. Meanwhile, the use of digital interviews in this study substantially facilitated data collection, widened the possibility to participate from the whole country rather than only locally, and may have facilitated recruitment of affected individuals who may not readily disclose their personal suffering and show up in person in a mental health–and addicion-oriented research unit. Indeed, challenges, but also potential advantages, of online qualitative research interview have been discussed in the research literature in recent years [37], pointing to the fact that such online interviews can play an important role in qualitative research.

Another potential limitation is the time aspect; some of the participants had recent or even ongoing experiences of Spelpaus, while others had been using it further back in time. Although this may be seen as a limitation, their information about their Spelpaus-related experience still is of great value (ie, as their ongoing or previous experience).

Additionally, the absence of a diagnosis of gambling gisorder as an inclusion criterion means that assessments of participants’ gambling problems relied solely on self-reported statements and estimations, which may not capture the full spectrum of gambling-related issues. This limitation underscores the possibility that experiences with Spelpaus may vary based on individuals’ level of gambling problems.

However, this study addressed a novel research issue and has strengths in a number of aspects. This is, to the best of our knowledge, the first qualitative study centered around the users’ experiences of the relatively novel and unique nationwide self-exclusion service. Consequently, it offers updated and in-depth insights into gamblers’ experiences with Spelpaus, elucidating its advantages and challenges as a harm reduction tool.

### Conclusion

This qualitative study aimed to deepen the knowledge about and understanding of a novel nationwide service for self-exclusion from gambling. This includes the experience of individuals who have a history of being self-excluded, including their reasons for self-exclusion and their experience of the system’s merits and challenges. Based on the interviews, this study suggests that although breaching one’s self-exclusion remains a challenge, users of this service also report major advantages from being self-excluded. Users suggest further improvements to the self-exclusion model, such as international collaboration in order to prevent overseas gambling operators offering gambling outside of the self-exclusion system and also structured links from self-exclusion to further help and support, possibly also involving formal treatment. The study provided new information about self-exclusion and about a broader ranges of reasons for self-excluding, over and above the reporting of severe gambling problems (eg, the role of self-exclusion in the relationship with concerned significant others or to prove willingness to quit gambling). Several favorable effects from self-excluding were reported, in addition to a need for clearer obstacles against going back to gambling once self-exclusion ends.
